# Golgi pH, Ion and Redox Homeostasis: How Much Do They Really Matter?

**DOI:** 10.3389/fcell.2019.00093

**Published:** 2019-06-11

**Authors:** Sakari Kellokumpu

**Affiliations:** Faculty of Biochemistry and Molecular Medicine, University of Oulu, Oulu, Finland

**Keywords:** homeostasis, Golgi pH, Golgi redox state, glycosylation, protein sorting, cancer

## Abstract

Exocytic and endocytic compartments each have their own unique luminal ion and pH environment that is important for their normal functioning. A failure to maintain this environment – the loss of homeostasis – is not uncommon. In the worst case, all the main Golgi functions, including glycosylation, membrane trafficking and protein sorting, can be perturbed. Several factors contribute to Golgi homeostasis. These include not only ions such as H^+^, Ca^2+^, Mg^2+^, Mn^2+^, but also Golgi redox state and nitric oxide (NO) levels, both of which are dependent on the oxygen levels in the cells. Changes to any one of these factors have consequences on Golgi functions, the nature of which can be dissimilar or similar depending upon the defects themselves. For example, altered Golgi pH homeostasis gives rise to *Cutis laxa* disease, in which glycosylation and membrane trafficking are both affected, while altered Ca^2+^ homeostasis due to the mutated SCPA1 gene in *Hailey–Hailey* disease, perturbs various protein sorting, proteolytic cleavage and membrane trafficking events in the Golgi. This review gives an overview of the molecular machineries involved in the maintenance of Golgi ion, pH and redox homeostasis, followed by a discussion of the organelle dysfunction and disease that frequently result from their breakdown. *Congenital disorders of glycosylation* (CDGs) are discussed only when they contribute directly to Golgi pH, ion or redox homeostasis. Current evidence emphasizes that, rather than being mere supporting factors, Golgi pH, ion and redox homeostasis are in fact key players that orchestrate and maintain all Golgi functions.

## What Is Golgi Homeostasis?

Compartmentalization is a key feature of eukaryotic cells, and it allows cells to complete various tasks with amazing speed and specificity. One drawback to compartmentalization is its need for the extra energy required to generate the unique luminal environments of each compartment. Unlike the ER, the other secretory pathway compartments, such as the ERGIC, Golgi apparatus and secretory vesicles, are all mildly acidic (Kim et al., 1996; Demaurex et al., 1998; Palokangas et al., 1998; Schapiro and Grinstein, 2000; Wu et al., 2001; Paroutis et al., 2004). Each of them has its own unique resting pH (pH set point) that facilitates their efficient functioning, be it membrane trafficking, cargo selection, glycosylation, proteolysis, or protein sorting. Nevertheless, these compartments also have common properties with the ER, with which they all communicate at least to some extent. The ER and the Golgi apparatus are the most closely associated compartments, sharing several common properties including high calcium concentration and oxidative potential, as well as the ability to synthesize glycans. This review, focusing on the Golgi apparatus, will discuss ER-related processes for comparative purposes only.

In general, organelle acidity is driven by the ATP-mediated proton pump, the V(vacuolar)-ATPase, which is counterbalanced by anion influx or cation efflux, and proton leak back to the cytoplasm via a “H^+^ leak channel” whose identity still remains elusive (Paroutis et al., 2004). Many other energy consuming pumps and leak channels, which are needed to maintain balanced Cl^-^, Ca^2+^, Mn^2+^ and K^+^ levels, are also present in the Golgi membranes. These include the Golgi pH regulator (GPHR, a chloride channel), a mid-1-related chloride channel (MClC) and voltage-gated chloride channels ClC-3B in mammalian cells, and Gef-1 in yeast (Schwappach et al., 1998; Jentsch et al., 1999; Nagasawa et al., 2001; Gentzsch et al., 2003; Maeda et al., 2008). Golgi membranes also contain two different isoforms of the Na^+^/H^+^ exchanger (NHE7 and NHE8), of which NHE7 seems to mediate the influx of Na^+^ or K^+^ in exchange for H^+^ (Numata and Orlowski, 2001; Lin et al., 2005). Although the exact physiological roles of many of these transporters remain unclear, they are known to contribute to Golgi resting pH, membrane potential, vesicular trafficking and protein sorting in the organelle. The use of fluorescent redox probes has recently revealed the Golgi redox state to be important for Golgi homeostasis and functions. Samoylenko et al. (2013) showed that the oxidative potential of the Golgi is higher than that of the endoplasmic reticulum (ER), the main site of disulfide bond formation in the cells. Indeed, earlier observations had shown that disulfide bonds can also form “late,” i.e., in the Golgi compartment, and facilitate disulfide bond-mediated oligomerization of some secretory products before their secretion to the extracellular space (Wagner, 1990; Walter et al., 2009; Chiu and Hogg, 2019). Cellular oxygen levels also regulate the level of Golgi nitric oxide (NO), a free radical that has been shown to be important for maintaining Golgi morphology and for ensuring continued membrane trafficking to the cell surface (Galkin et al., 2007; Nakagomi et al., 2008; Lee et al., 2011, 2013). Taken together, these examples highlight the complexity of factors needed to maintain the unique Golgi environment and the functions that depend on it. The following paragraphs summarize the molecular machineries involved, before focusing on why their failures result in organelle dysfunction and disease.

## Regulation of Golgi pH, Ion and Redox Homeostasis

### Transport of Protons and Golgi pH Homeostasis

The acidity of the Golgi lumen was first demonstrated in 1983 by using electron microscopy and a compound (DAMP) that accumulated in acidic cellular compartments (Glickman et al., 1983). Currently, several different fluorescence-based approaches have been used to identify proteins that contribute to Golgi acidity and its resting pH (Glickman et al., 1983; Kim et al., 1996, Kim et al., 1998; Demaurex et al., 1998; Llopis et al., 1998; Miesenbock et al., 1998; Schapiro and Grinstein, 2000; Wu et al., 2000, 2001; Machen et al., 2001; Paroutis et al., 2004). These studies have shown that different Golgi sub-compartments have distinct pH set points, decreasing along the *cis–trans* axis of the Golgi stack from pH 6.7 (*cis*-Golgi) to pH 6.0 at the *trans*-Golgi network (TGN). A pertinent question is how this pH gradient is established and maintained along the Golgi stack, given its dynamic nature resulting from the continuous flow of incoming and leaving vesicular carriers. Another related issue is whether a similar gradient also applies to other ions that are uniquely concentrated in the Golgi lumen and thus contribute to its ion homeostasis.

The resting pH of the Golgi lumen is now known to be determined mainly by three different ion transport systems that include the vacuolar (V)-ATPase-mediated proton pump, counter ion (Cl^-^) transport, and proton “leak” across the Golgi membranes back to the cytoplasm (Wu et al., 2001; Demaurex, 2002; Paroutis et al., 2004). In brief, the V-ATPase uses ATP as an energy source to pump protons into the Golgi lumen. Due to proton pumping, the membrane potential starts to increase (inside positive) and must be counterbalanced by Cl^-^ influx. This very likely takes place via the GPHR Cl^-^ channel (or a cation efflux channel). Once the Golgi pH is sufficiently acidic (pH < 6.3), proton efflux via an elusive “proton leak channel” prevents further acidification of the Golgi lumen. The resting pH, or the pH set point, is established once the rate of proton pumping matches its leak rate across Golgi membranes. Because of continuous H^+^ pumping by the V-ATPase, it is the rate of H^+^ leakage that dictates the resting pH of the organelle (Wu et al., 2001). The authors showed that the rate of proton efflux decreases between successive secretory compartments. They also suggested that the higher density of H^+^ pumps in the later secretory compartments may also contribute to their lower resting pH. These two factors are likely responsible also for the decreasing pH gradient along the *cis*- to *trans*-axis of the Golgi stacks, even though direct proof for this does not yet exist.

**Figure 1:**
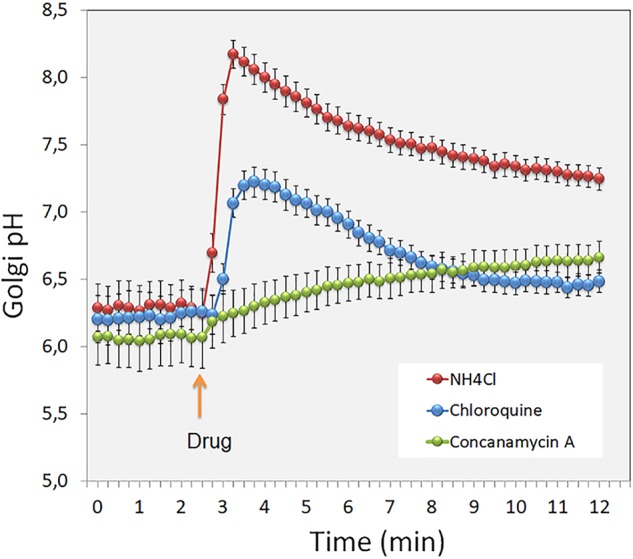
The figure shows short term (min) changes in the Golgi luminal pH after treating intact cells with the pH gradient dissipating agents (red and blue dots) and the V-ATPase inhibitor Concanamycin A (green dots). Note the differential pH responses to these drugs, and the rate of H^+^ leakage across the Golgi membranes after shutting down the V-ATPase by the inhibitor used.

The V-ATPase itself is a multi-subunit protein complex (Drory and Nelson, 2006; Jefferies et al., 2008), whose composition may vary between different compartments. For example, the Golgi localized V-ATPase appears to possess a subunit *a* different from that found in other V-ATPases (the Stv1p instead of the Vph1p in yeast) (Jefferies et al., 2008). The V-ATPase activity is also regulated by glucose or nutrient levels, yet under normal conditions (i.e., at least when counter-ion conductance is sufficient and, therefore, does not restrict proton pumping), it is assumed to be constantly active (Schapiro and Grinstein, 2000; Wu et al., 2001). In support of this, the Golgi lumen in intact cells starts to alkalinize when the V-ATPase activity is shut down by using concanamycin A (Figure 1, green dots).

Cl^-^ influx seems to be normally required to prevent membrane potential increase due to proton pumping by the V-ATPase (Glickman et al., 1983; Schapiro and Grinstein, 2000; Paroutis et al., 2004). Under normal conditions, it is considered to be high enough and mediated by the GPHR protein channel termed the Golgi pH Regulator (Maeda et al., 2008). Mutation of the protein was shown to increase Golgi resting pH (by 0.4–0.5 pH units), alter glycosylation, delay transport to the plasma membrane, and induce Golgi fragmentation. These findings thus provide strong support for the view that H^+^ pumping is dependent on Cl^-^ influx and is needed to maintain a constant membrane potential. The extent to which other Golgi-localized chloride channels, such as the voltage-gated chloride channels ClC-3B (Gentzsch et al., 2003) and Gef1p in yeast (Schwappach et al., 1998) regulate Golgi resting pH remains unclear.

Other studies have suggested that continuous H^+^ pumping may be facilitated by passive K^+^ efflux rather than by Cl^-^ influx (Howell and Palade, 1982). This may relate to a high permeability of the Golgi membranes to K^+^ ions (Schapiro and Grinstein, 2000), and could perhaps be mediated by Na^+^ and K^+^ conductive channels or transporters such as the Na^+^/K^+^-ATPase (Poschet et al., 2001). In support of the latter possibility, acetylstrophanthidin (an inhibitor of the Na^+^/K^+^-ATPase) was proposed to increase luminal acidity by inhibiting electrogenic Na^+^/K^+^ exchange (3 Na^+^ for 2 K^+^), thereby reducing the accumulation of other cations (relative to H^+^) in the Golgi lumen. Alternatively, the Na^+^/H^+^ exchanger NH7 could also facilitate the acidification of the Golgi lumen by transporting H^+^ into the Golgi lumen in exchange for luminal K^+^ ions (Numata and Orlowski, 2001). However, recent data indicates that NH7 does not transport K^+^ ions (Milosavljevic et al., 2014), thus leaving open whether Na^+^ ions may suffice for an acid loading function of this exchanger in the Golgi compartment.

### Proton Leak Across the Golgi Membranes

Despite its importance, the identity of the “proton leak channel” still remains elusive. It may involve exchange of luminal protons for cytosolic cations via a proton conductive channel, or via import of base equivalents. Physiological measurements indicate that proton efflux in the TGN is voltage-sensitive and inhibited by Zn^2+^, suggesting the involvement of a regulated channel (Cherny and DeCoursey, 1999; Schapiro and Grinstein, 2000). Other studies suggest that the molecular characteristics of a putative H^+^ channel mimic those of the plasma membrane H^+^ channels (Numata and Orlowski, 2001; Nakamura et al., 2005). Therefore, the two ubiquitously expressed, Golgi-localized Na^+^/H^+^ exchanger isoforms, NHE7 and NHE8, are good candidates for this channel, because Na^+^/H^+^ exchange is normally driven by existing ion gradients, and high amounts of H^+^ in the Golgi lumen will drive influx of Na^+^. In support of this, overexpression of both NHE7 and NHE8 were found to increase Na^+^ and K^+^ influx to the Golgi lumen (Numata and Orlowski, 2001), and both also raised the Golgi resting pH (Nakamura et al., 2005). However, changes in sodium concentration during Golgi pH measurements did not markedly alter the Golgi resting pH (Demaurex et al., 1998), leaving some doubts about the possible roles of these NHEs in mediating proton leakage across Golgi membranes. In accordance with this, Milosavljevic et al. (2014) recently showed that NHE7, at least when expressed at the plasma membrane, acts as an acid loader rather than as a “H^+^ leak” pathway in the cells. The NHE8 isoform also seems to have more pronounced effects on endosomes than it has on the Golgi (Lawrence and Bowers, 2010). Therefore, further work is needed to clarify the exact roles of the NHEs in the Golgi membranes.

**Figure 2:**
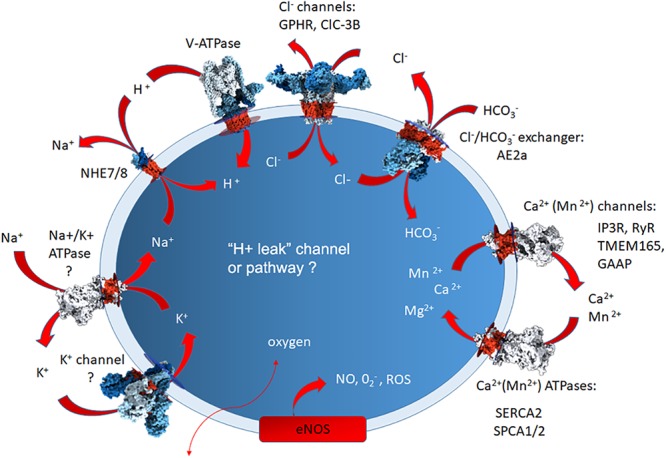
A simplified summary of the Golgi-localized ion pumps, transporters and channels identified at the protein level, or based upon Golgi physiological measurements. The question marks depict those for which direct proof for the presence of the protein in question needs to be verified. The arrows depict their putative direction of functioning in ion transport. The elusive H+ leak channel is also marked by question mark to depict the lack of knowledge about its identity. For additional details, see the text.

Soluble buffering molecules may also be used for regulating the Golgi resting pH. In support of this view, a homolog of the erythrocyte anion exchanger 1 (Band 3, AE1, SLC4A1) was identified as the AE2a isoform (SLC4A2a) of the SLC4A gene family in the Golgi membranes in a number of cell types (Kellokumpu et al., 1988; Holappa et al., 2001). All members of this gene family are electroneutral HCO_3_^-^/Cl^-^ exchangers, regulating cytosolic pH, chloride concentration and cell volume through an obligatory one to one exchange of chloride for bicarbonate (Romero et al., 2004; Alper, 2006). Our recent high-throughput Golgi pH measurements indicate that it is involved in Golgi pH regulation, as its overexpression increased, and knockdown decreased, the Golgi resting pH (Khosrowabadi et al., unpublished). The potential involvement of various channels, pumps and transporters in the maintenance of Golgi homeostasis is summarized in Figure 2.

### Transport of Ca^2+^ and Mn^2+^ Ions and Golgi Homeostasis

Ca^2+^, Mg^2+^, or Mn^2+^ ions are present at high concentrations in the Golgi lumen (Van Baelen et al., 2004; Pizzo et al., 2010). Their presence is important for cargo concentration and sorting (Chanat and Huttner, 1991) and glycosylation (Leach, 1971; Vanoevelen et al., 2007). Golgi membranes also possess relevant pumps for Ca^2+^ and Mn^2+^ uptake (SERCA2, SPCA1/2), channels for Ca^2+^ release (IP3R, RyR) and luminal proteins that bind Ca^2+^ with high affinity (Van Baelen et al., 2004; Brini and Carafoli, 2009; Lin et al., 2009; Vangheluwe et al., 2009; Zampese and Pizzo, 2012). Mn^2+^ ions are important cofactors for Golgi-resident glycosyltransferases. The DXD motif conserved in many glycosyltransferases appears to have a key role in Mn^2+^-mediated donor substrate binding and catalytic activity (Breton et al., 2006). Mn^2+^ ions also act as scavengers for reactive oxygen species (ROS) (Coassin et al., 1992). Of these, SERCA and SPCA type pumps are responsible for the maintenance of the low cytosolic and high luminal Ca^2+^ concentrations typical of many secretory pathway compartments. These two Ca^2+^ pumps seem to contribute differentially to Ca^2+^ uptake into the Golgi, as SERCA2 is enriched in the *cis*-Golgi, while SPCA1 is mainly present in the *trans*-Golgi (Wong et al., 2013). The localization of Ca^2+^ release channels, the inositol-1,3,5-trisphosphate receptor (IP3Rs) and the ryanodine receptor (RyR), also seems to be different, as IP3 did not release Ca^2+^ in the *trans*-Golgi, while activation by caffeine did so (Vanoevelen et al., 2004; Lissandron et al., 2010; Wong et al., 2013). Contrasting with SERCAs, SPCAs are also engaged in Mn^2+^ transport, and thus can provide this essential trace metal supply to Golgi glycosyltransferases (Vangheluwe et al., 2009).

Recent evidence also suggests that mutations of TMEM165 cause a *type II congenital disorder of glycosylation* in humans by interfering with Mn^2+^ and, perhaps, also Ca^2+^/H^+^ transport (Foulquier et al., 2012; Dulary et al., 2017, 2018; Thines et al., 2018) and, therefore, also with Golgi ion and pH homeostasis and glycosylation. However, it is not yet fully clear what role this multi-spanning membrane protein plays in Golgi ion homeostasis. Recent evidence indicates that, unlike the Golgi-localized SPCA1, the ER-associated SERCA pump 2b isoform partially rescued TMEM165 KO-induced glycosylation defect by Mn^2+^ (Houdou et al., 2019). Moreover, those authors also recently showed that the TMEM165 KO can also be rescued by galactose supplement in HEK293 cell culture media, or when given to patients (Morelle et al., 2017). Further studies are needed to reveal the exact role of the TMEM165 transporter in the maintenance of Golgi ion homeostasis.

Another multi-spanning membrane protein capable of transporting Ca^2+^ has been identified in the Golgi membranes. This protein, named Golgi-associated anti-apoptotic protein (GAAP), has recently gained increasing attention due to its role in tumorigenesis (Rojas-Rivera and Hetz, 2015; Carrara et al., 2017). It is a member of the Transmembrane Bax Inhibitor-1 Motif-containing (TMBIM) protein family regulating Ca^2+^ levels and fluxes in intracellular stores, confering resistance to a broad range of apoptotic stimuli and promoting cell adhesion and migration via the activation of store-operated Ca^2+^ entry (SOCE) (Saraiva et al., 2013; Carrara et al., 2015).

The Golgi lumen also harbors several Ca^2+^-binding proteins, including Cab45, CALNUC, p54/NEFA and calumenin, all of which, except for Cab45, are distinct from their ER counterparts. Of these, the most abundant is CALNUC, an EF-hand, Ca^2+^-binding protein resident in the CGN and *cis*-Golgi cisternae. It plays a major role in Ca^2+^ buffering and secretion through the Golgi (Lin et al., 1999, 2009). Recent studies of Cab45 have demonstrated its importance in Ca^2+^-mediated protein sorting. Cab45 is the core component of this oligomerization-driven sorting mechanism, also involving the cytoplasmic actin cytoskeleton, and the Ca^2+^ ATPase SPCA1 (Pakdel and von Blume, 2018). The system relies first on the local synthesis of sphingomyelin at the TGN membrane enhancing Ca^2+^ import by SPCA1, which then drives secretory protein sorting and export, thereby coupling lipid synthesis to protein sorting and secretion (Deng et al., 2018).

### Golgi Redox Homeostasis

Genetically encoded and targeted fluorescent probes such as roGFP and HyPer have been used to determine organelle redox states (Meyer and Dick, 2010; Lukyanov and Belousov, 2014). By using roGFP2 as a probe, we have previously shown that mitochondria have a less oxidizing environment than that of the ER (Samoylenko et al., 2013). Intriguingly, it was also found that the Golgi lumen is more oxidizing than the ER despite being considered as the most oxidizing compartment in eukaryotic cells. One possibility for its higher oxidizing power is that it serves for “late” disulfide bond formation, as indicated in studies showing that the assembly of von Willebrand factor oligomers to multimers, or other secretory products, requires tail-to-tail disulfide bond formation in the Golgi (Wagner, 1990; Chiu and Hogg, 2019). Such disulfide bond formation in the Golgi is likely assisted by members of the Quiescin-Sulfhydryl Oxidase (QSOX) gene family (Codding et al., 2012) that all display PDI-like thioredoxin (Trx) domains and ERV-like oxidase domains. These domains allow QSOX proteins to efficiently couple disulfide bond formation with the reduction of molecular oxygen to hydrogen peroxide. QSOX1 is widely expressed within the secretory pathway compartments, while one of the splice variants, QSOX1a, mainly localizes to the Golgi (Chakravarthi et al., 2007; Heckler et al., 2008). This suggests its involvement in disulfide bond formation, possibly related to the maturation of ECM components, or to the formation of higher order structures in the Golgi. Other proteins that can regulate Golgi redox homeostasis include the glutaredoxins Grx6 and Grx7 (Mesecke et al., 2008). They both belong to an ubiquitous family of proteins that catalyze the reduction of disulfide bonds with the help of reduced glutathione. Grx6 and Grx7 represent the first glutaredoxins found in the *cis*-Golgi in baker’s yeast (Mesecke et al., 2008). They both show a high glutaredoxin activity *in vitro*, and yeast cells lacking both proteins exhibit growth defects and a strongly increased sensitivity toward oxidizing agents. Grx6 and Grx7 are probably important for counteracting oxidation-driven disulfide bond formation in the Golgi.

The availability of oxygen is also intimately linked to the Golgi redox state. When low, it causes hypoxia, a condition that affects multiple cellular compartments including mitochondria and the ER. Recent evidence indicates that hypoxia also modulates Golgi functions and, in particular, those related to membrane trafficking and glycosylation events. Accordingly, hypoxia has been shown to alter expression levels of both glycosyltransferase and nucleotide sugar transporter genes, and to inhibit membrane trafficking between the ER and the Golgi (Koike et al., 2004; Shirato et al., 2011; Belo et al., 2015; Bensellam et al., 2016; Taniguchi et al., 2016). We recently showed that hypoxia modulates the Golgi redox state and glycosylation without markedly affecting Golgi pH homeostasis (Hassinen et al., 2019; see also below). Oxygen levels not only affect the redox state of the Golgi lumen, but also the production of NO levels by modulating the activity and expression of various NO synthase isoforms, including neuronal nitric oxide synthase (nNOS), inducible NOS (iNOS), and endothelial NOS (eNOS) (Jeffrey Man et al., 2014). Of these, only eNOS is located to the Golgi membranes (Iwakiri et al., 2006) via myristylation or palmitylation of its N-terminus. NO is a lipophilic compound and can readily pass through membranes. Both NO and superoxide (O_2_^-^), another possible product of eNOS activity, are highly reactive free radicals and increase ROS load.

## Altered Golgi Homeostasis in Golgi Dysfunction and Disease

### Membrane Trafficking and Protein Sorting Defects

Failure to maintain Golgi pH, ion, and redox homeostasis is commonly associated with membrane trafficking and protein sorting defects. Monensin, a Na^+^/H^+^ ionophore, was the first compound shown to block intra-Golgi transport between the medial- and *trans*-Golgi cisternae (Griffiths et al., 1983a,b). Kuismanen et al. (1985) reported that intracellular transport of the Uukuniemi virus membrane glycoproteins (G1 and G2) was not inhibited by monensin. Whether this discrepant behavior is cell or virus type-dependent remains unclear. Protein sorting in the Golgi is also dependent upon existing pH gradients. In line with these observations, Schaub et al. (2006) showed that monensin induces the relocalization of B4GalT1 galactosyltransferase (but not ST6Gal-I) and alpha-1,3-fucosyltransferase 6 (Schaub et al., 2008) in swollen vesicles derived from the TGN based on their colocalization with TGN46, a specific TGN marker. This relocalization was also found to be signal-mediated, involving a short sequence in its cytoplasmic tail, which, when present in ST6Gal-I, was able to relocate the latter into the TGN-derived swollen vesicles from the *trans*-Golgi cisternae or the TGN. However, the signals were not needed for the steady state localization of these enzymes in the *trans*-Golgi cisternae.

A better example of pH-sensitive membrane trafficking steps is the retrograde transport from the Golgi back to the ER. This was demonstrated using bafilomycin A (a V-ATPase specific inhibitor), which inhibited retrograde, but not anterograde, transport from the intermediate compartment (IC)/*cis*-Golgi back to the ER (Palokangas et al., 1998). This preferential effect on retrograde trafficking may relate to the more acidic environment at the IC/*cis*-Golgi interface than that of the ER and to the pH-dependent retrieval system mediated by the KDEL-receptor (see below). Based on these observations, it seems that cargo selection and membrane fission is more sensitive to a pH change than membrane fusion is, thereby inhibiting or delaying transport between successive secretory compartments., However, due to the pH-sensitivity of viral protein-mediated membrane fusion events with endosomal membranes (White and Whittaker, 2016; Desai et al., 2017), this scenario needs further testing. Indeed, these viral fusion events typically occur via “inside to outside” fusions with organelle membranes and are therefore topologically opposite from fusions that take place between vesicular carriers and their target membranes.

Another well-characterized example of a pH-dependent protein sorting step is the KDEL receptor, which returns escaped ER resident proteins from the *cis*-Golgi back to the ER. The receptor is a key component of a homeostatic control system that regulates trafficking between the ER and the Golgi compartments and within the Golgi itself (Lewis and Pelham, 1992; Scheel and Pelham, 1996; Cancino et al., 2014). The receptor binds the peptide sequence KDEL (or a similar sequence motif), leading to interaction with two different Golgi-associated heterotrimeric G-proteins, which regulate the transport machineries via phosphorylation (Giannotta et al., 2012; Cancino et al., 2014). Of notice here is that both cargo binding and its release are regulated by the pH gradient between the two organelles (see Brauer et al., 2019, and references therein). In the more acidic environment of the *cis*-Golgi, the receptor recognizes the motif and binds to it, while at the neutral pH of the ER lumen, it releases the motif and the associated cargo. Moreover, p58/ERGIC-53/LMAN1, a receptor protein involved in the export of soluble glycoproteins from the ER, employs a similar pH gradient for its oligomerization and accessory protein-mediated binding with specific cargo and its release in the low pH-high calcium environment at the ER-Golgi interface (Appenzeller-Herzog et al., 2004; Appenzeller-Herzog and Hauri, 2006).

A third well-known example is the mannose-6-phosphate receptor, which binds lysosomal enzymes carrying the man-6-P tag in the Golgi and releases them in the lower pH environment of the endosomes (Ghosh et al., 2003). In line with this, we showed that, in some cancer cell lines with problems in lysosomal acidification, the ligand-bound receptor cannot unload its ligand in lysosomes and accumulates in endosomal/lysosomal compartments (Kokkonen et al., 2004). This suggested that further lysosomal enzyme cargo sorting at the TGN is impossible and can result in their aberrant secretion into the extracellular space, a phenomenon that is often associated with tumorigenesis and likely helps cancer cells to invade and metastasize to adjacent tissues (Mohamed and Sloane, 2006; Gocheva and Joyce, 2007; Kallunki et al., 2013).

Golgi pH homeostasis is also important for the sorting of apical and basolateral proteins in polarized epithelial cells. Caplan et al. (1987) showed that laminin and heparan sulfate proteoglycan (HSPG) are normally actively sorted to the basolateral surface of polarized canine renal tubule cells (MDCK) in a pH-dependent manner. By increasing the pH of the Golgi and other cellular compartments in MDCK cells with NH_4_Cl (Figure 1, red dots), the authors were able to divert the two above-mentioned secretory proteins to both the apical and basolateral transport vesicles, with the outcome that roughly equal amounts were sorted to both surfaces in the treated cells. Since the TGN is the main sorting station for these two surface domains (Guo et al., 2014), it is likely that the cargo recognition and sorting at the TGN may not depend only on specific sorting signals but also on the existence of an environment suitable for their recognition by the sorting machinery in each case. We recently showed that the apical targeting of the CEAMCAM5 (carcinoembryonic antigen, CEA), a well-known follow-up marker for colorectal cancer, is also a pH-sensitive process (Kokkonen et al., 2018). CEA is a typical GPI-anchored apical protein present in gut epithelial cells. For an unknown reason, CEA exhibits a non-polarized distribution in cancer cells, such as in CaCo-2 cells. Guided by the notion that the Golgi resting pH is ∼0.5 pH units higher in CaCo-2 cells than in non-malignant cells, we treated MDCK cells stably expressing CEA with various compounds, including concanamycin A (CMA: a proton pump inhibitor, see Figure 1). We showed that, in contrast to drugs affecting the redox state, CMA attenuated apical targeting of CEA without affecting its trafficking to the cell surface. In the presence of the drug, CEA was delivered equally to apical and basolateral domains of MDCK cells due to inhibition of its GPI anchor-mediated association with lipids rafts.

Autosomal recessive Cutis Laxa type II is the first inherited disease identified thus far that is tightly linked to altered Golgi pH homeostasis. The skin of these patients shows excessive wrinkling at an early age. It is caused by mutations in the gene encoding the *a*2 subunit of the Golgi-localized V-ATPase (*ATP6V0A2*) (Kornak et al., 2007). However, patients belonging to a closely related disease group suffering from Wrinkly skin syndrome are heterogeneous, in that only some patients carry the same mutation and show no symptoms of elastin deficiency (Morava et al., 2009). Although the Golgi resting pH has not yet been directly measured, it is expected that this V-ATPase defect perturbs Golgi pH homeostasis, because the patients’ cells exhibited glycosylation and membrane trafficking defects (Morava et al., 2005; Kornak et al., 2007; Hucthagowder et al., 2009). Further studies are still needed, as Golgi membranes also seem to co-express another subunit, the “*a*1” isoform, of the V-ATPase (see Kornak et al., 2007). Nevertheless, the “*a*2” subunit mutations impair retrograde trafficking from the Golgi back to the ER, but here again, the mechanistic details remain unclear. One complicating factor in gaining an understanding of the *cutis laxa* phenotype is the fact that the “*a*2” subunit appears to localize also in early endosomes (Hurtado-Lorenzo et al., 2006), suggesting that altered endosomal pH and dysfunction may also contribute to the disease etiology.

Khayat et al. (2019) recently described a new pH homeostasis-associated disease with multigenerational non-syndromic intellectual disability (ID). The disease is caused by missense mutations in the alkali cation/proton exchanger NHE7 (SLC9A7). The variant protein localized correctly in the TGN/post-Golgi vesicles, but its *N*-linked glycans were abnormal likely due to less acidic pH of the TGN/post-Golgi compartments in patient’s cells. Membrane trafficking, however, was unaffected. These observations are consistent with a role for NHE7 in the regulation of TGN/post-Golgi pH homeostasis and suggest that abnormal Golgi pH homeostasis may be the cause of neurodevelopmental defects associated with this disease.

Other luminal ions, and particularly Ca^2+^, also contribute to membrane trafficking defects. The depletion of cellular Ca^2+^ stores in NRK cells using thapsigargin abolished KDEL receptor-mediated retrieval of ER chaperones GRP94/endoplasmin and GRP78/BiP, resulting in their appearance in the culture medium (Ying et al., 2002). Accordingly, thapsigargin was found to inhibit Brefeldin A-induced retrograde transport from the Golgi back to the ER in HeLa cells (Ivessa et al., 1995). Calcium depletion also selectively inhibited proteolytic cleavage of pro-somatostatin or proinsulin, without affecting their secretion (Austin and Shields, 1996). It also interfered with the sorting of secretogranin II into immature granules in semi-intact PC12 cells (Carnell and Moore, 1994), even though high Ca^2+^ and low pH have been reported to facilitate the concentration of cargo proteins in regulated secretory vesicles (Chanat and Huttner, 1991).

SPCA1 is a Golgi-localized Ca^2+^ ATPase that transports both Ca^2+^ and Mn^2+^ into the Golgi lumen and, therefore, plays an important role in Golgi cation homeostasis (Van Baelen et al., 2004; Missiaen et al., 2007). In humans, allelic mutations of the SPCA1 gene (Vanoevelen et al., 2007; Brini and Carafoli, 2009) are the cause of Hailey–Hailey disease, in which patients’ keratinocytes exhibit an increased cytosolic Ca^2+^ concentration, and defects in protein sorting and Ca^2+^ signaling (Missiaen et al., 2004; Ramos-Castaneda et al., 2005; Vanoevelen et al., 2007). Lowered levels of Ca^2+^ and Mn^2+^ cations in the Golgi lumen in patients’ cells lead to defects in protein folding, trafficking and sorting or proteolytic cleavage of prohormones (Missiaen et al., 2004; Grice et al., 2010). These defects could explain why the affected patients are unable to maintain structurally intact desmosomes and epidermis. The fact that Mn^2+^ is an important cofactor for many glycosyltransferases (Kaufman et al., 1994) suggests that glycosylation is altered in affected cells and may also contribute to the disease etiology.

Golgi Ca^2+^ (and Mn^2+^) homeostasis is also dependent on cellular oxygen levels. A good example of this is the fact that intermittent hypoxia upregulates the expression of both SPCAs in HCT116 cells (Jenkins et al., 2016), suggesting that Ca^2+^ and/or Mn^2+^ transport from the cytosol to the Golgi lumen via SPCAs likely increases in hypoxic cells. Oxygen also regulates nitrogen oxide (NO) levels in the Golgi by modulating eNOS activity and thus, NO production, thereby locally enhancing S-nitrosylation of Golgi proteins, especially of the *N*-ethylmaleimide-sensitive factor (NSF) (Iwakiri et al., 2006). Since NSF is involved in membrane fusion events, this modification delays protein transport from the ER to the plasma membrane and, thus, can at least partially explain why hypoxia inhibits ER-Golgi vesicular trafficking. On the other hand, compounds that can scavenge NO (such as c-PTIO, *N*-acetylcysteine and hemoglobin) induced Golgi fragmentation (Lee et al., 2011, 2013), which was accompanied by the depletion of α-soluble NSF acceptor protein (α-SNAP) from Golgi membranes, in accordance with the observed delay in ER-Golgi trafficking.

### Golgi pH Homeostasis and Glycosylation Defects

Glycosylation is likely the most pH-sensitive process of the Golgi functions. For example, monensin was shown to prevent processing of Uukuniemi viral G proteins into endo-H-resistant and under-sialylated species without affecting membrane trafficking (Kuismanen et al., 1985). Campbell et al. (2001) in turn were able induce the expression of oncofetal Thomsen-Friedenreich (TF- or T-) antigen in LS174T goblet-differentiated cells by increasing Golgi pH with bafilomycin A and monensin. Axelsson et al. (2001) were the first to provide a mechanistic link for these pH-induced glycosylation changes by using prolonged NH_4_Cl treatment in HeLa and LS 174T cells. They showed that inhibition of *O*-glycan synthesis by NH4Cl was accompanied by mis-localization of *N*-acetylgalactosaminyltransferase 2, b-1,2-*N*-acetylglucosaminyltransferase I and b-1,4-galactosyltransferase 1 into endosomal compartments, while the drug had no effect on Golgi morphology. However, because most of the enzymes that elongate *O*-glycans were not addressed in the study, it remains unclear whether enzyme re-localization is solely responsible for the observed glycosylation defect(s). Later, by using increasing concentrations of chloroquine, we (Rivinoja et al., 2006) demonstrated that only a 0.2 pH unit increase in Golgi luminal pH is needed to interfere with mucin type *O*-glycosylation and terminal a-2,3-sialylation of *N*-linked glycans without causing any changes to overall Golgi morphology. The latter defect correlated well with the observed mislocalization of the relevant sialyltransferase (ST3Gal-III) into endosomal compartments, while no such redistribution was observed with ST6Gal-I (or B4GalT-I), i.e., the enzyme that adds sialic acid to terminal galactose residue via an a-2,6-linkage. *Cutis laxa* type II patients also display defects in sialylation of both *N*-linked and *O*-linked glycans (Morava et al., 2005; Wopereis et al., 2005; Kornak et al., 2007). These observations indicate that glycosylation in general is highly sensitive to changes in Golgi luminal pH and, if altered, can be due to mislocalization of a selected set of glycosyltransferases. Other known causes are changes in the expression levels of the enzymes, yet these do not strictly correlate with glycan profiles displayed by the cells.

Most glycosyltransferases show an intrinsic tendency to form oligomeric complexes with each other. Typically, such complexes include enzymes that successively add sugar residues to a glycan chain (Kellokumpu et al., 2016). All enzymes also form homomers in the ER (Hassinen and Kellokumpu, 2014), perhaps facilitating their folding or transport to the Golgi, or both. On the other hand, enzyme heteromers only form after the enzymes arrive in the Golgi compartment. This switch from enzyme homomers to enzyme heteromers is dependent on the pH gradient or Golgi redox homeostasis (see section “Golgi Redox Homeostasis and Altered Glycosylation” last paragraph) between the ER and the Golgi. Thus, heteromer formation of *N*-glycan processing enzymes B4GalT-I and ST3Gal-III, and of enzymes that synthesize mucin type *O*-glycan core structures (ppGalNacT-6, C1GalT-1, C2/3GNT), is prevented by increasing Golgi pH with chloroquine (Hassinen et al., 2011). Intriguingly, the same enzymes (except B4GalT-I) were also found to mislocalize in chloroquine treated cells (unpublished observations), suggesting that heteromer formation may contribute to their retention or retrieval in the Golgi membranes. However, in other cases such as B4GalT-I and ST6Gal-I, heteromer formation was not affected by an increase in Golgi luminal pH (Hassinen et al., 2011), but rather by an altered Golgi redox state (Hassinen et al., 2019; see also Section “Golgi Redox Homeostasis and Altered Glycosylation” last paragraph). These observations point to fundamental differences in the way enzyme heteromers form in the Golgi lumen, and perhaps reflecting the high specificity of the interactions needed to prevent irrational interactions that could otherwise lead to the synthesis of mixed or irrelevant glycan structures (Kellokumpu et al., 2016). The high specificity for the interactions could also explain a failure to identify any consensus Golgi retention motif(s) in Golgi enzymes, except those needed for their retrieval from later compartments to earlier ones via GOLPH3-mediated binding to the COPI complex (Rabouille and Klumperman, 2005; Tu et al., 2008; Sechi et al., 2015; Liu et al., 2018). Whether such Golgi retention motifs involving relevant enzyme interactions in different Golgi sub-compartments indeed exist remains to be tested. However, their existence is supported by the pH-dependent mislocalization of a set of glycosyltransferases (Rivinoja et al., 2009; unpublished observations); otherwise, it would be difficult to understand how luminal alkalization can interfere with the recognition of retrieval motifs on the cytosolic side of the Golgi membranes. In addition, oligomerization that inherently involves enzyme interactions, has been considered to be important for the retention of resident glycosyltransferases in the Golgi (Nilsson et al., 2009).

The loss of enzyme heteromers is generally accompanied by changes in *O*- and *N*-linked glycosylation, but likely concerns other glycosylation pathways as well. One reason for these changes is that heteromer formation significantly increases the activity of the complex constituents (Kellokumpu et al., 2016). For example, both enzyme activities of B4GalT-I/ST6Gal-I heteromers were 2.5-fold higher than their respective homodimers (Hassinen et al., 2011). How this activation is achieved is currently unclear but may involve substrate channeling or conformational changes brought about by the interaction. In other words, the formation of the enzyme heteromers from enzyme homomers in the Golgi could simply serve to keep enzymes silent until they arrive in the Golgi. Such a system would increase both the speed and fidelity of glycan synthesis, as it would also prevent the intervention of competing enzymes that can use the same acceptor sugar as a substrate. This view is in line with the pH-independent α-2,6-sialylation and formation of B4GalT-I/ST6Gal-I heteromers (in contrast to α-2,3-sialylation and the formation of ST3Gal-III/B4GalT-I heteromers; see Rivinoja et al., 2009; Hassinen et al., 2011). The difference in pH-dependency can also explain why the carcinoembryonic antigen (CEACAM5) extracted from colon cancer tissue carries α-2,6-linked sialic acid instead of the α-2,3-linked sialic acid found in normal tissues (Yamashita et al., 1987; Kobata et al., 1995). The extent to which the loss of enzyme heteromers contributes to glycosylation remains to be determined when enzyme interaction mutants become available.

### Cancer-Associated Glycosylation Changes

Altered glycosylation is one of the hallmarks of cancers. Such alterations can involve changes in the elongation of *O*-glycans, the branching of *N*-glycans, sulfation, *O*-acetylation of sialic acid, fucosylation and the expression of blood group antigens (Jass et al., 1994; Kuhns et al., 1995; Capon et al., 1997; Taylor-Papadimitriou et al., 1999; Hakomori, 2002; Roth, 2002; Lau and Dennis, 2008; Ungar, 2009; Reis et al., 2010; Radhakrishnan et al., 2014; Vajaria and Patel, 2017; Rodrigues and Macauley, 2018). Some of these changes are used as cancer markers, while others also have verified roles in promoting tumorigenesis (Hakomori, 1991, 1996; Ono and Hakomori, 2004; Peixoto et al., 2016). Based on existing data, several factors that can cooperatively contribute to the above cancer-associated glycosylation changes have been put forward. These include the altered expression of glycosyltransferases or nucleotide sugar transporter genes (Yang et al., 1994; Brockhausen et al., 1995; Hanisch et al., 1996; Lloyd et al., 1996; Kumamoto et al., 2001), and perhaps also a loss of activity of Cosmc, a specific molecular chaperone needed for the folding and catalytic activation of C1GalT-I (Schietinger et al., 2006). The C1GalT-I enzyme normally adds galactose to the Tn-antigen (GalNAc-Ser), forming a mucin type *O*-glycan core 1 structure (the T-antigen). Thus, the loss of its activity may result in an increased expression of the Tn-antigen in cancer cells.

Cancer-associated glycosylation changes can also result from a more general defect related to altered Golgi ion or pH homeostasis. The first observations suggesting this came from studies in which the treatment of cells with pH gradient dissipating drugs increased the expression of cancer-associated Tn- and T-antigens (Thorens and Vassalli, 1986; Gawlitzek et al., 2000; Axelsson et al., 2001; Campbell et al., 2001; Kellokumpu et al., 2002). At high concentrations, these same compounds also induced Golgi fragmentation typically seen also in cancer cells (Kellokumpu et al., 2002). Direct Golgi pH measurements with fluorescent probes in breast and colorectal cancer cells (MCF-7, HT-29, SW-48) showed that the Golgi resting pH is indeed more alkaline (∼0.2–0.4 pH units) than that of non-malignant cells (Rivinoja et al., 2006). These early observations strongly supported the view that abnormally high Golgi resting pH is responsible for the increased expression of cancer-associated glycan antigens.

An important question at the time was why abnormal Golgi pH is detrimental to glycosylation. Although one still cannot exclude possible effects on the synthesis or transport of nucleotide sugars, we believe that either the loss of the *O*-glycosyltransferase heteromers or enzyme mislocalization, or both, are the two main reasons for the pH-dependent glycosylation changes seen in cancer cells. These two factors might in fact also be interlinked, given that oligomerization appears to be important for Golgi retention (Nilsson et al., 1993, 1994, 2009). Thus, at elevated Golgi resting pH, the enzymes responsible for synthetizing the *O*-glycan core structure are unable to form heteromers (Hassinen et al., 2011). Their loss then abrogates their retention in the Golgi, whereby they mislocalize to endosomal compartments and are therefore unable to elongate the core GalNAc residue with other sugar residues in the Golgi. In support of this view, *O*-glycosyltransferases seem to have altered distribution in cancer cells *in vivo* (Egea et al., 1993). Similar relocalization of the initiating ppGalNAcT-1/2 to the ER was seen after growth factor-induced activation of the src kinase, or by transfecting cells with constitutively active src (Gill et al., 2010, 2011). This relocalization was linked to a COP-I-dependent trafficking event, as a dominant-negative Arf1 isoform, Arf1(Q71L), blocked ppGalNacT redistribution. Note however, that the pH-induced relocalization of the enzyme involved transport to the endosomal compartments via bulk flow (Rivinoja et al., 2009), suggesting that pH-induced relocalization of the enzymes is associated with impaired Golgi retention, rather than with activated transport from the Golgi to the ER.

Another issue related to organelle acidification defects in cells is its association with the multidrug resistance (MDR). In certain MDR cancer cell lines, chemotherapeutic drugs (often weak bases) become protonated and sequestered in acidic organelles (Schindler et al., 1996; Altan et al., 1998). Sequestration in resistant cells allows the removal of cytotoxic drugs from the cytoplasm via secretory and recycling pathways. In contrast, drug-sensitive cells were shown to have defects in organelle acidification, whereby a similar sequestration of the drugs does not occur, exposing the cells to high concentrations of the drugs. However, these acidification defects are not universal in all MDR cells, suggesting that other mechanisms for MDR exist (Simon, 1999).

### Golgi Redox Homeostasis and Altered Glycosylation

Reactive oxygen species and hypoxia (low oxygen environment) are key modulators of the cellular redox state (Adler et al., 1999). ROS and hypoxia also modulate Golgi-associated vesicular trafficking, protein sorting and glycosylation events (Regoeczi et al., 1991; Koike et al., 2004; Yin et al., 2006; Shirato et al., 2011; Ermini et al., 2013; Lehnus et al., 2013; Belo et al., 2015). Most often, this is thought to be mediated mainly by hypoxia-inducible factors (HIF-1-3) that regulate the expression of hundreds of genes, including a variety of proteins involved in glycosylation. Specifically, hypoxia has been shown to down- or up-regulate enzymes that synthetize nucleotide sugars in the cytoplasm, Golgi-localized glycosyltransferases (Mgat2, Mgat-3 and Mgat5 and 5b, fucosyltransferases 1, 2 and 7, sialyltransferases ST3Gal-I and ST6Gal-1) and transporters of UDP-Galactose, CMP-sialic acid (Sialin) and UDP-*N*-acetylglycosamine (UGT1) (Koike et al., 2004; Shirato et al., 2011; Belo et al., 2015; Taniguchi et al., 2016). Some of these are involved in the synthesis of cancer-associated sialyl Lewis A/X carbohydrate epitopes typically found in selectins on *O*-linked glycans and glycolipids (Kumamoto et al., 2001; Shirato et al., 2011). HIF-1α in the Pa-Tu-8988S and Pa-Tu-8988T pancreatic cancer cell lines have also been shown to suppress the expression of the UDP-glucuronosyltransferase (Kato et al., 2016), cytosolic *O*-GlcNAc transferase (OGT) (Liu et al., 2014) and glucosylceramide synthase (GCS) (Zhao et al., 2003). Finally, Jenkins et al. (2016) showed that expression of the SPCA2 Ca^2+^ pump in HCT116 colon cancer cells was upregulated by hypoxia, and by reactive oxygen and nitrogen species. The authors suggested that this upregulation is associated with Mn^2+^-dependent cell cycle arrest, but whether these changes relate to increased Ca^2+^ or Mn^2+^ transport to the Golgi lumen, Ca^2+^-mediated protein sorting, glycosylation, or detoxification from excess Mn^2+^, remains unclear.

Based on the above observations, Taniguchi et al. introduced the term “Glyco-redox” to link altered glycosylation with oxidative stress generated by hypoxia or ROS (Taniguchi et al., 2016), and to emphasize their close association with Parkinson disease, Alzheimer’s disease, amyotrophic lateral sclerosis (ALS), and chronic obstructive pulmonary disease (COPD). These changes may also partly involve cleavage of cell surface glycosaminoglycans and *N*-linked glycans, thereby affecting interactions of cells with the extracellular matrix (Eguchi et al., 2002; Eguchi et al., 2005), as has been observed using hypoxia-mimicking agents such as CoCl_2_ (Taniguchi et al., 2016). Oxidative stress also provides a link between altered glycosylation, high-fat diet and the onset of type II diabetes (Ohtsubo and Marth, 2006; Ohtsubo, 2010; Ohtsubo et al., 2011). High levels of free fatty acids were shown to inhibit the activity of two transcription factors (Foxa2 and Hnf1a) that normally positively regulate the expression of Mgat4a, a glycosyltransferase needed for β1,4-GlcNAc branching of *N*-glycans. This modification is needed for insulin-stimulated transport of the GLUT-4 transporter to the cell surface, whereby it is able to bind and import glucose for further use by the pancreatic β-cells. In the absence of β1,4-GlcNAc branching, the GLUT-4 transporter remains intracellular, leading to an accumulation of glucose in the blood.

We have recently shown that hypoxia also modulates glycosylation in a HIF-independent manner by reducing Golgi redox state (Hassinen et al., 2019). Specifically, we demonstrated that even moderate hypoxia (5% O_2_) lowers the Golgi oxidizing potential to the level found in the ER of normoxic cells, consistent with possible problems in disulfide bond formation. Based on lectin microarray glycan profiling, this decrease was accompanied by an attenuated sialylation of *N*-glycans, an elongation of *O*-linked glycans, the loss of pH-independent interaction between the B4GalT-I and ST6Gal-I, and the loss of ST6Gal-I activity in hypoxic cells. These findings can explain the reduced a-2,6-sialylation in hypoxic cells. Given that sialylation was not the only change in glycosylation, we expect that other enzymes are similarly affected by a lowered Golgi oxidizing potential.

## Concluding Remarks

The above examples highlight the critical roles of Golgi pH, ion and redox homeostasis in the maintenance of Golgi functions and its unique architecture. The existing data also emphasizes that Golgi acidity, high cation concentrations and redox state are not just background components in the Golgi, but rather, they emerge as the key players in orchestrating various membrane trafficking events, keeping the Golgi resident enzymes correctly localized and active, and facilitating their cooperative interactions in glycosylation. In addition, they are important for cargo selection by post-Golgi vesicular carriers and for protein sorting to the apical surface in polarized epithelial cells. Further support of their importance is provided by several human diseases, including *Cutis Laxa, Hailey–Hailey disease* and *congenital disorder of glycosylation* 2K (CDG2K), which all are caused by altered ion homeostasis in the Golgi lumen. Other Golgi homeostasis-associated diseases are certainly waiting to be identified.

Although many mechanistic details remain incompletely understood, the current evidence indicates that each of the Golgi pH, ion or redox (oxygen) regulatory systems can perturb all Golgi functions simultaneously or may specifically impair only one of them. For example, a small increase in Golgi luminal pH (0.2 pH units) will perturb glycosylation with no detectable effect on other Golgi functions. A failure to maintain Golgi oxidative potential causes defects in both membrane trafficking and glycosylation, with no detectable changes in enzyme localization in the Golgi. High Ca^2+^ concentrations seem to contribute to membrane trafficking and protein sorting events at the Golgi, but also to glycosylation, since many enzymes need Mn^2+^ ions to remain catalytically active.

The above examples highlight the complexity and mutual interplay of the regulatory systems needed to establish and maintain Golgi homeostasis and the many different cellular phenotypes encountered by manipulating homeostatic machineries with drugs or mutations. For example, it is well known that perturbed membrane trafficking often results in changes in Golgi architecture and glycosylation. The inter-dependence of these phenomena is probably the biggest obstacle for better understanding the role of each of these regulatory systems in the Golgi. Nevertheless, such studies are needed to provide us with new insights into how the various Golgi tasks are executed and to identify the molecular machineries that act in the background to keep these tasks ongoing. These studies will eventually unveil how the Golgi compartment functions as an organelle and what purpose(s) its unique architecture with stacked and flattened cisternae actually serves for.

## Data Availability

The datasets generated for this study are available on request to the corresponding author.

## Author Contributions

SK wrote the text and made the figures.

## Conflict of Interest Statement

The author declares that the research was conducted in the absence of any commercial or financial relationships that could be construed as a potential conflict of interest.
